# Publisher Correction: Compensatory ion transport buffers daily protein rhythms to regulate osmotic balance and cellular physiology

**DOI:** 10.1038/s41467-021-26725-7

**Published:** 2021-11-24

**Authors:** Alessandra Stangherlin, Joseph L. Watson, David C. S. Wong, Silvia Barbiero, Aiwei Zeng, Estere Seinkmane, Sew Peak Chew, Andrew D. Beale, Edward A. Hayter, Alina Guna, Alison J. Inglis, Marrit Putker, Eline Bartolami, Stefan Matile, Nicolas Lequeux, Thomas Pons, Jason Day, Gerben van Ooijen, Rebecca M. Voorhees, David A. Bechtold, Emmanuel Derivery, Rachel S. Edgar, Peter Newham, John S. O’Neill

**Affiliations:** 1 MRC Laboratory of Molecular Biology, Cambridge, UK; 2 Faculty of Biology, Medicine and Health, University of Manchester, Manchester, UK; 3 UCSF, San Francisco, CA USA; 4 California Institute of Technology, Pasadena, CA USA; 5 Department of Chemistry, University of Geneva, Geneva, Switzerland; 6 LPEM - ESPCI Paris, PSL, CNRS, Sorbonne Université, Paris, France; 7 Department of Earth Sciences, University of Cambridge, Cambridge, UK; 8 School of Biological Sciences, University of Edinburgh, Edinburgh, UK; 9 Department of Infectious Diseases, Imperial College London, London, UK; 10 Clinical Pharmacology and Safety Sciences, R&D, AstraZeneca, Cambridge, UK; 11 Present Address: Crown Bioscience Netherlands B.V., Utrecht, The Netherlands; 12 Present Address: CEA, IRIG, SyMMES, Grenoble, France

**Keywords:** Proteome, Circadian rhythms, Cardiovascular biology

Correction to: *Nature Communications* 10.1038/s41467-021-25942-4, published online 15 October 2021.

The wrong Supplementary file was originally published with this article; it has now been replaced with the correct file. The original article has been corrected.

## Supplementary information

Updated Supplementary information

