# Supplementary material for: Compensatory ion transport buffers daily protein rhythms to regulate osmotic balance and cellular physiology
Source: Nat Commun. 2021 Oct 15;12:6035. doi: 10.1038/s41467-021-25942-4 (PMC8520019; doi:10.1038/s41467-021-25942-4)
Supplement: Supplementary file 1 — Supplementary information [file 41467_2021_25942_MOESM1_ESM.pdf]

Supplementary information

Compensatory ion transport buffers daily protein rhythms to regulate osmotic balance and cellular physiology

Authors: Alessandra Stangherlin[1], Joseph L. Watson[1], David C. S. Wong[1], Silvia Barbiero[1], Aiwei Zeng[1], Estere Seinkmane[1], Sew Peak Chew[1], Andrew D. Beale[1], Edward A. Hayter[2], Alina Guna[3], Alison J. Inglis[4], Marrit Putker[1††], Eline Bartolami[5†], Stefan Matile[5], Nicolas Lequeux[6], Thomas Pons[6], Jason Day[7], Gerben van Ooijen[8], Rebecca M. Voorhees[4], David A. Bechtold[2], Emmanuel Derivery[1], Rachel S. Edgar[9], Peter Newham[10], John S. O'Neill[1*].

Affiliations:
[1]MRC Laboratory of Molecular Biology, Cambridge, UK.

[2]Faculty of Biology, Medicine and Health, University of Manchester, Manchester, UK.

[3]UCSF, San Francisco, USA.

[4]California Institute of Technology, Pasadena, USA.

[5]Department of Chemistry, University of Geneva, Switzerland.

[6]LPEM - ESPCI Paris, PSL, CNRS, Sorbonne Université, Paris, France.

[7]Department of Earth Sciences, University of Cambridge, Cambridge, UK.

[8]School of Biological Sciences, University of Edinburgh, Edinburgh, UK.

[9]Imperial College London, UK.

[10]Clinical Pharmacology and Safety Sciences, R&D, AstraZeneca, Cambridge, UK.

*Correspondence to: oneillj@mrc-lmb.cam.ac.uk

† Current address: CEA, IRIG, SyMMES, Grenoble, France.

†† Current address: Crown Bioscience Netherlands B.V., Utrecht, The Netherlands.

Supplementary Fig. 1

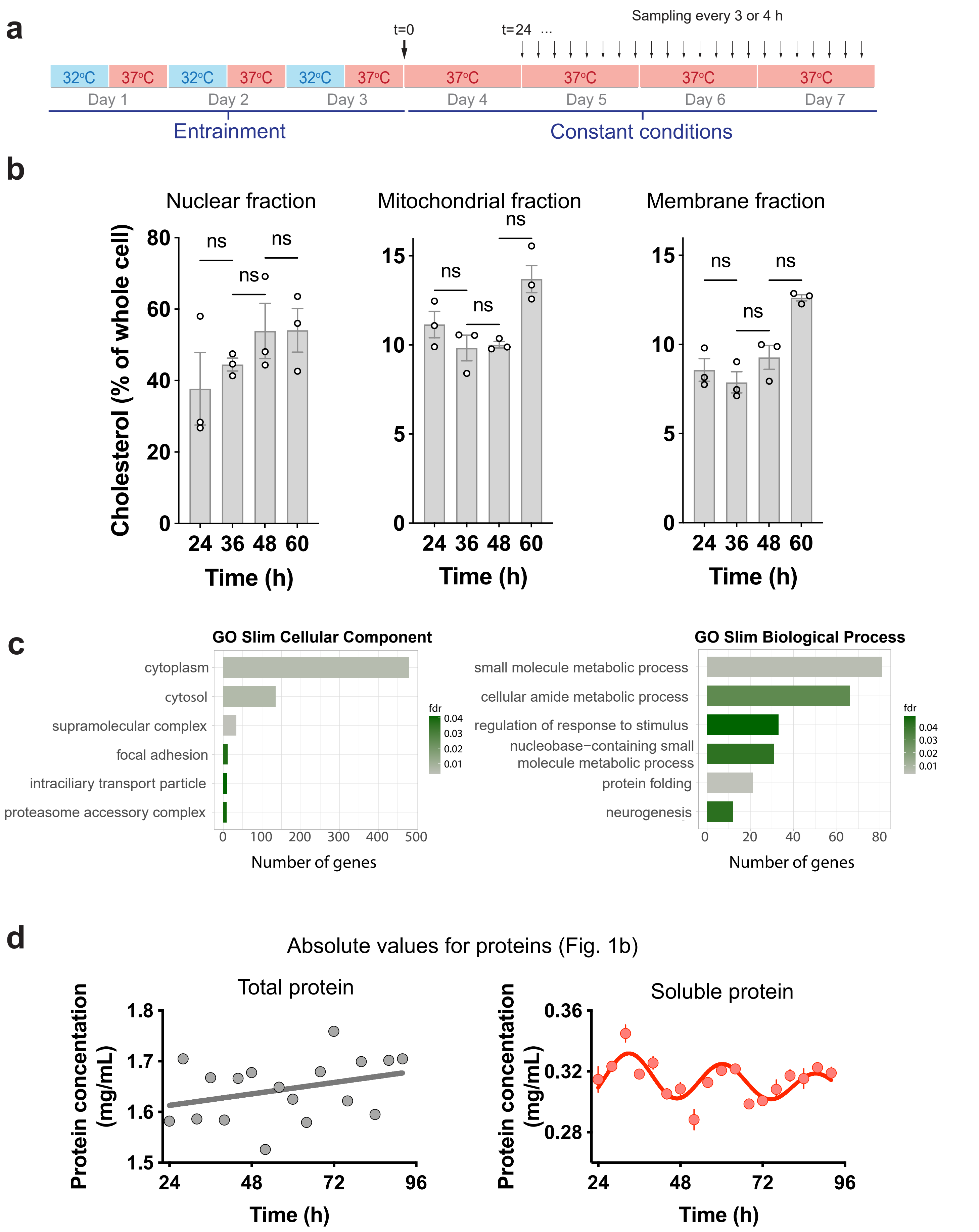

Supplementary Fig. 1. **Gene ontology analysis of digitonin lysates.**

a Schematic of time course experiments. **b** Cholesterol abundance in subcellular fractions at indicated times (n=3). These data exclude time-of-day variation in the extraction efficiency of digitonin that, in principle, might have arisen from a circadian rhythm of membrane cholesterol abundance. Significance was calculated using one-way ANOVA and Tukey's multi comparisons test (MCT). **c** Gene ontology analysis on digitonin extracts (n=4) using PANTHER enrichment test. Shown are top 6 terms for GO-Slim Cellular Component and GO-Slim Biological Process annotation data categories. Mann-Whitney U test and Benjamini-Hochberg correction (FDR, $p<0.05$) were performed. **d** Absolute values for soluble and total protein presented in Fig. 1b. p-values indicate comparison of damped cosine wave with straight-line fit (null hypothesis = no rhythm) (n=3).

Mean ± SEM shown throughout. Cell type used in (**b-d**) was lung fibroblasts.

Supplementary Fig. 2

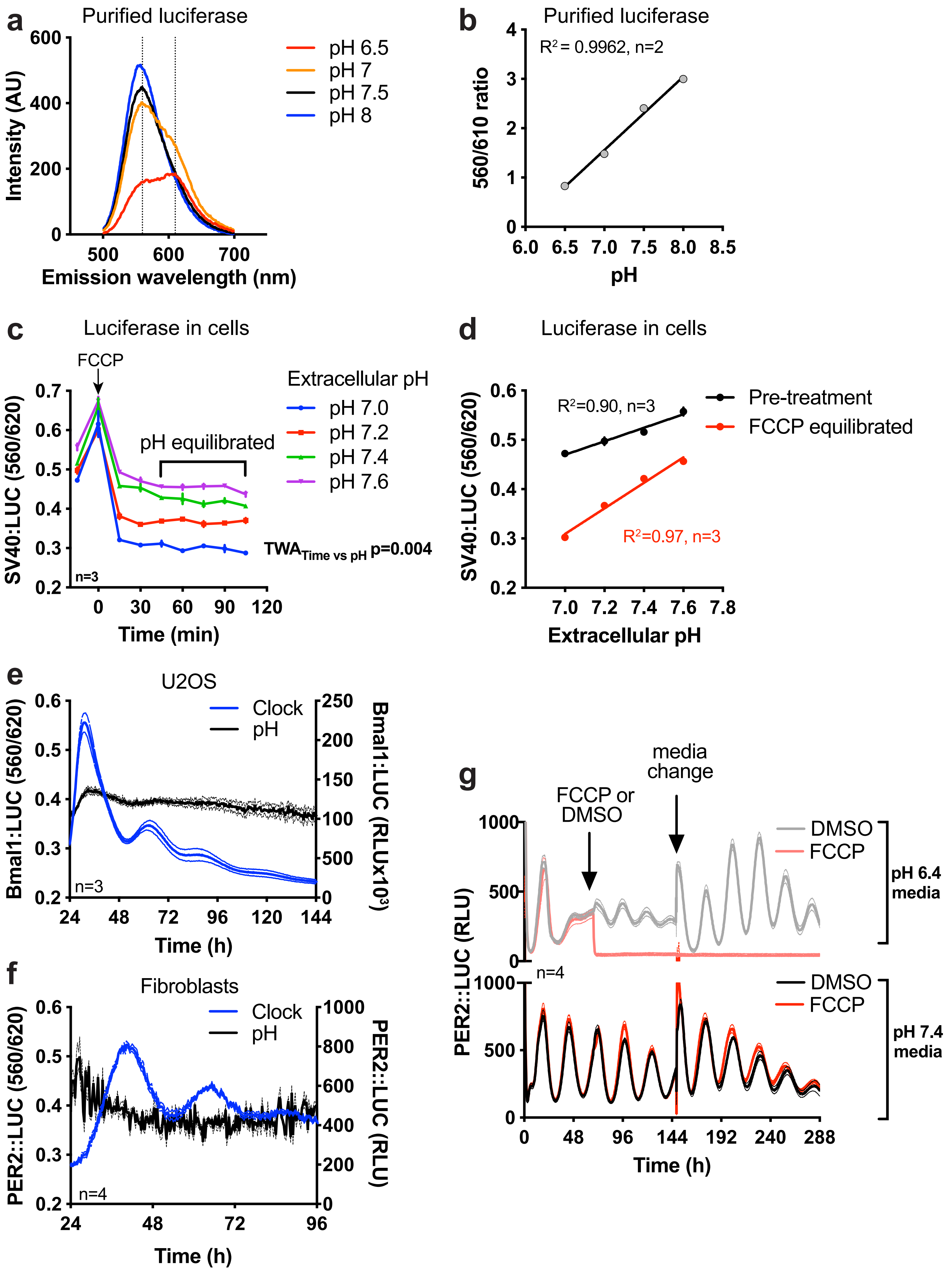

Supplementary Fig. 2. **Cytosolic pH measurements.**

Use of firefly luciferase (Fluc) as a ratiometric pH reporter demonstrates no circadian rhythm in cytosolic pH. **a** Luminescence emission spectra of purified luciferase solutions at different pH, confirming the pH-sensitivity of native Fluc[102]. **b** 560nm/610nm emission ratio plotted as a function of pH. **c** Validation of Fluc as a pH sensor in U2OS cells expressing Fluc constitutively (SV40:LUC), where cells were incubated with media of different pH (7.0-7.6) which has only a modest effects on basal cytosolic pH. Following treatment with FCCP (a proton ionophore), intracellular pH equilibrates with extracellular pH, with a commensurate shift in 560 nm/620 nm ratio. **d** 560/620 emission ratio plotted as a function of pH, confirming a linear relationship between pH and the 560 nm/620 nm ratio of Fluc bioluminescence in living cells (n=3). **e, f** Representative traces showing gene expression rhythms and pH measured in human U2OS cells and mouse lung fibroblasts using the Bmal1:LUC transcriptional reporter (**e**) or PER2::LUC translational reporter (**f**). Whilst circadian gene expression rhythms were observed in total bioluminescence of both reporters, no such oscillation in pH was observed. This contrasts with data collected using exactly the same method during the yeast respiratory oscillation, where cytosolic pH varies consistently and was robustly detected by the ratio of Fluc bioluminescence emmission[45]. This demonstrates that overall cytosolic pH does not consistently vary over the circadian cycle of cultured mammalian cells but leaves open the possibility that daily variation in highly localised pH transients might fulfil some specific timing function. **g** We tested this in PER2::LUC lung fibroblasts (n=4) under sustained treatment with FCCP or DMSO (vehicle). FCCP treatment was clearly effective, since it was cytotoxic to cells with media of pH 6.4 (no recovery of bioluminescence rhythms after a media change, compared with DMSO controls). In contrast, addition of FCCP to cells with media of pH 7.4 had no effect on the period or amplitude of PER2::LUC rhythms, or on cell viability. Therefore, cytosolic pH is not circadian regulated and does not regulate circadian rhythms. Data are shown as mean ± SEM.

Supplementary Fig. 3

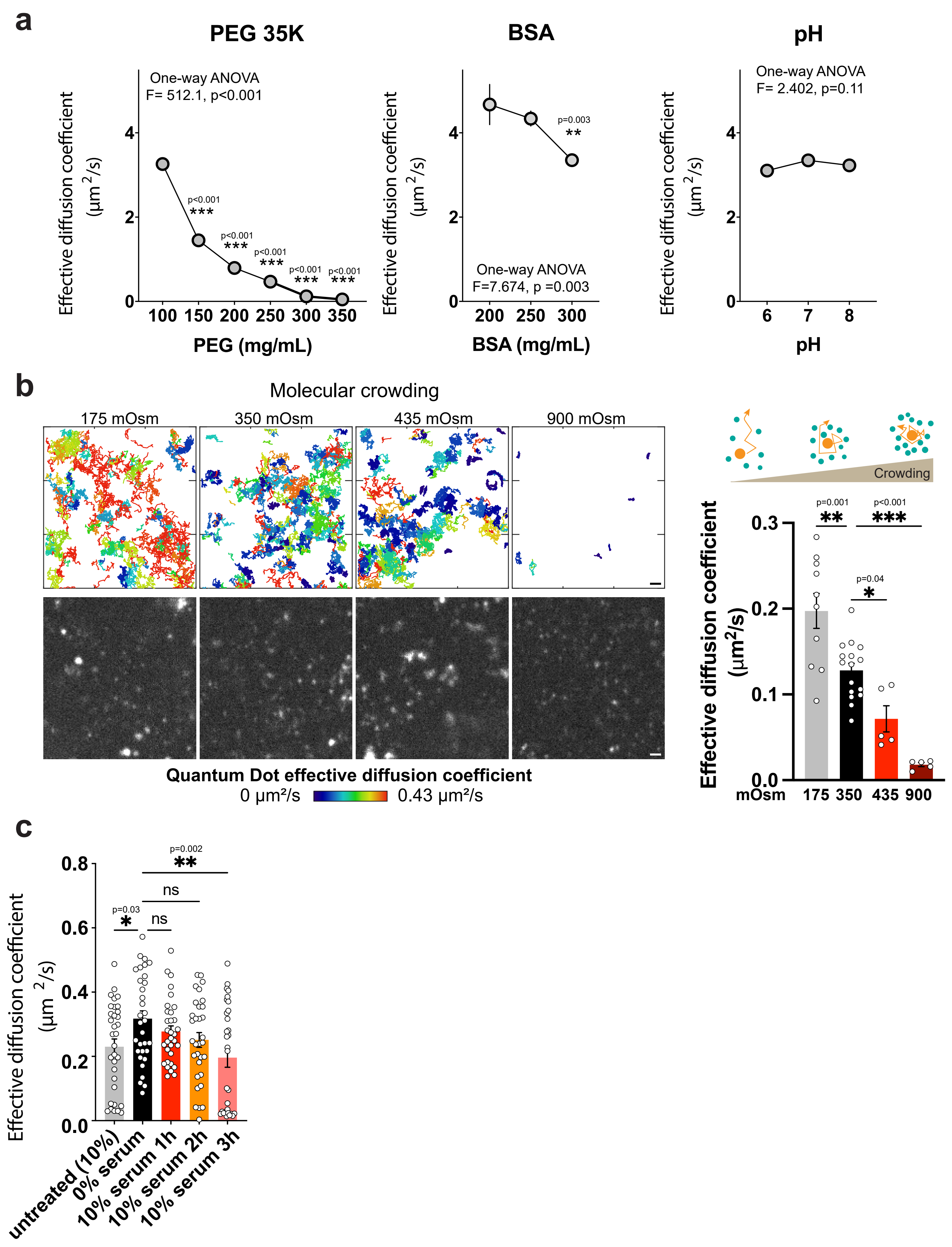

Supplementary Fig. 3. **Validation of QDs as a method to measure cytosolic macromolecular crowding.**

The diffusion of macromolecules in solution is affected by many factors, including pH, temperature, and size, but the most physiologically-relevant determinant is macromolecular crowding. **a** Measurement of the effective diffusion quantum dots (QDs) in solution. Diffusion of QDs is sensitive to increasing concentrations of macromolecules, such as polyethylene glycol (PEG 35K) (n= 8, 10, 10, 10, 10, 10 fields of view per concentration from 100 to 350 mg/mL) and bovine serum albumin (BSA, 300 mg/mL) (n=7, 10, 10 fields of view, respectively) but is not sensitive to changes in physiological pH (n=10 fields of view). **b** Validation of diffusion quantification from the effective diffusion rate of quantum dots (QDs) in fibroblasts upon treatment with media of different osmolality (n=10, 16, 5, 5) and representative tracking images. Scale bar: 1µm. **c** Effective diffusion coefficient of QDs in untreated cells and cells stimulated for the indicated time with 10% serum after starvation (n= 32, 32, 32, 30, 33).

Data are presented as Mean ± SEM. Statistical test used was one-way ANOVA with Dunnet's MCT. Cell type used was cardiac fibroblasts for (**b**) and lung fibroblasts for (**c**).

Supplementary Fig. 4

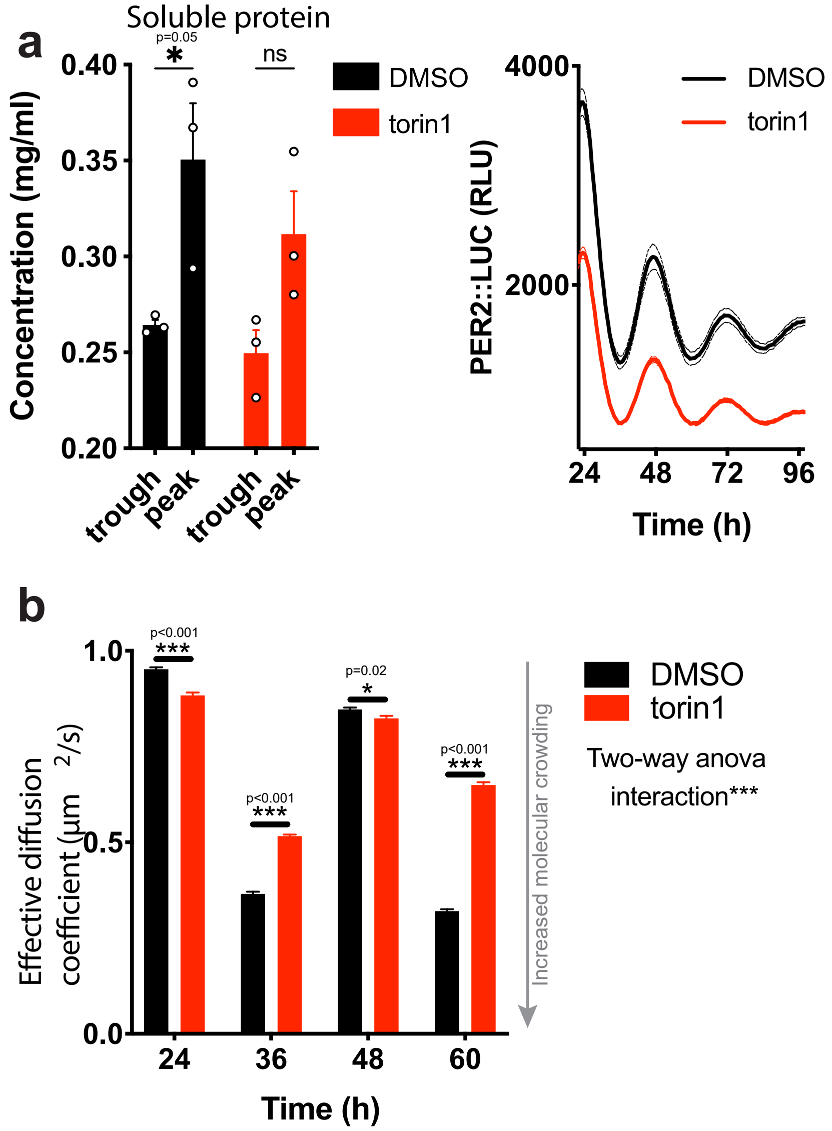

Supplementary Fig. 4. **mTORC inhibition attenuates soluble protein rhythms and time of day variation in the diffusion coefficient of Quantum Dots.**

a Quantification of soluble protein (n=3) in fibroblasts ± 50 nM torin1 at peak and trough of protein rhythms and bioluminescence data of the circadian clock reporter (n=3). **b** Effective diffusion rate of QDs in fibroblasts ± 50 nM torin1 (n= 57, 39, 49, 46 for DMSO and n=47, 57, 46, 37 for torin1). For both (**a**) and (**b**) torin1 was added to the medium at time 0 h and kept for the duration of the experiment.

Data are presented as mean ± SEM. Statistical significance calculated using one-way ANOVA with Sidak's MCT in (**a**) and two-way ANOVA in (**b**). Cell type used was lung fibroblasts.

Supplementary Fig. 5

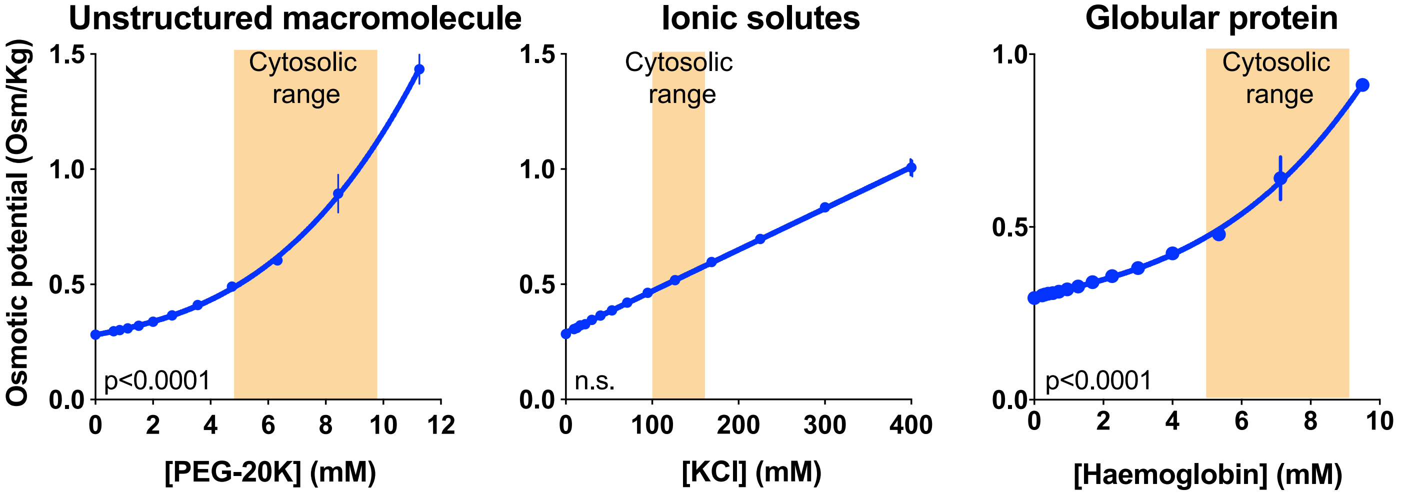

Supplementary Fig. 5. **Relationship between concentration and osmolality of macromolecules and ionic solutes.**

Osmolality of PEG-20K, KCl, and haemoglobin solutions (n=3 technical replicates). Unlike KCl, the crowding agent PEG20K and haemoglobin exhibit a non-linear relationship between concentration and osmolality due to increasing proportions of solvent molecules being affected by the extended hydration shells around macromolecules, compared with simple hydration shells of small ionic solutes[103–106]. We note that other interpretations for this phenomenon have been proposed[107]. At high concentrations, small changes in macromolecule concentration (unstructured and globular proteins) trigger much larger changes in osmolality than ionic solutes

Data are presented as mean ± SD. Non-linear fit of a third order polynomial was applied to PEG and haemoglobin datasets.

Supplementary Fig. 6

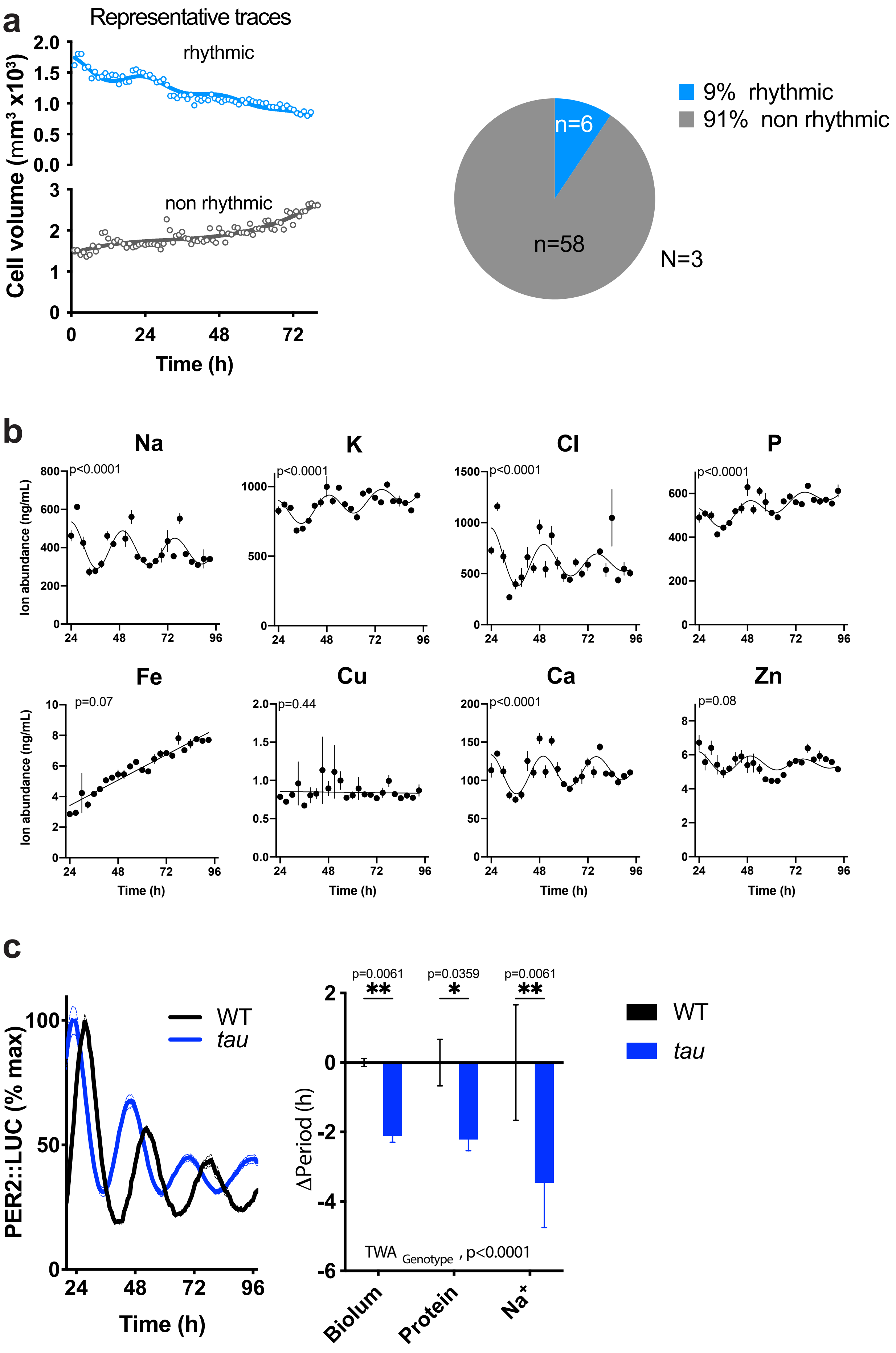

Supplementary Fig. 6. **Ion rhythms in fibroblasts occur with no volume change.**

a Representative measurements and quantification of fibroblast volume over several circadian cycles reveals that most (91%) cells show no significant circadian variation in volume. While feeding-dependent diurnal variation in hepatocyte volume was recently reported[6], only a small proportion (9%) of individual fibroblasts showed significant ~24 h variation in volume i.e. where a damped sine wave fit was preferred ($p<0.05$) over a straight-line fit (null hypothesis = no rhythm), and the period between 20 and 28 h. Moreover, the relative amplitude of these few cells that showed circadian variation was much lower than changes in ion content. Thus, changes in cellular volume cannot account for the daily variations in ion and cytosolic protein abundances we observe. **b** ICP-MS quantification of biologically-relevant ions in primary fibroblasts (n=4). p-values indicate comparison between a damped cosine wave fit compared with a straight-line (null hypothesis, no rhythm). **c** Bioluminescence control for WT and tau mutant fibroblasts. Comparison of ΔPeriod for clock reporter, soluble protein rhythms, and Na^+ rhythms (n=3). Statistical test used was 2-way ANOVA and Holm-Sidak's MCT.

Data are presented as mean ± SEM. Cell type used was cardiac fibroblasts for (**a, b**) and lung fibroblasts for (**c**).

Supplementary Fig.7

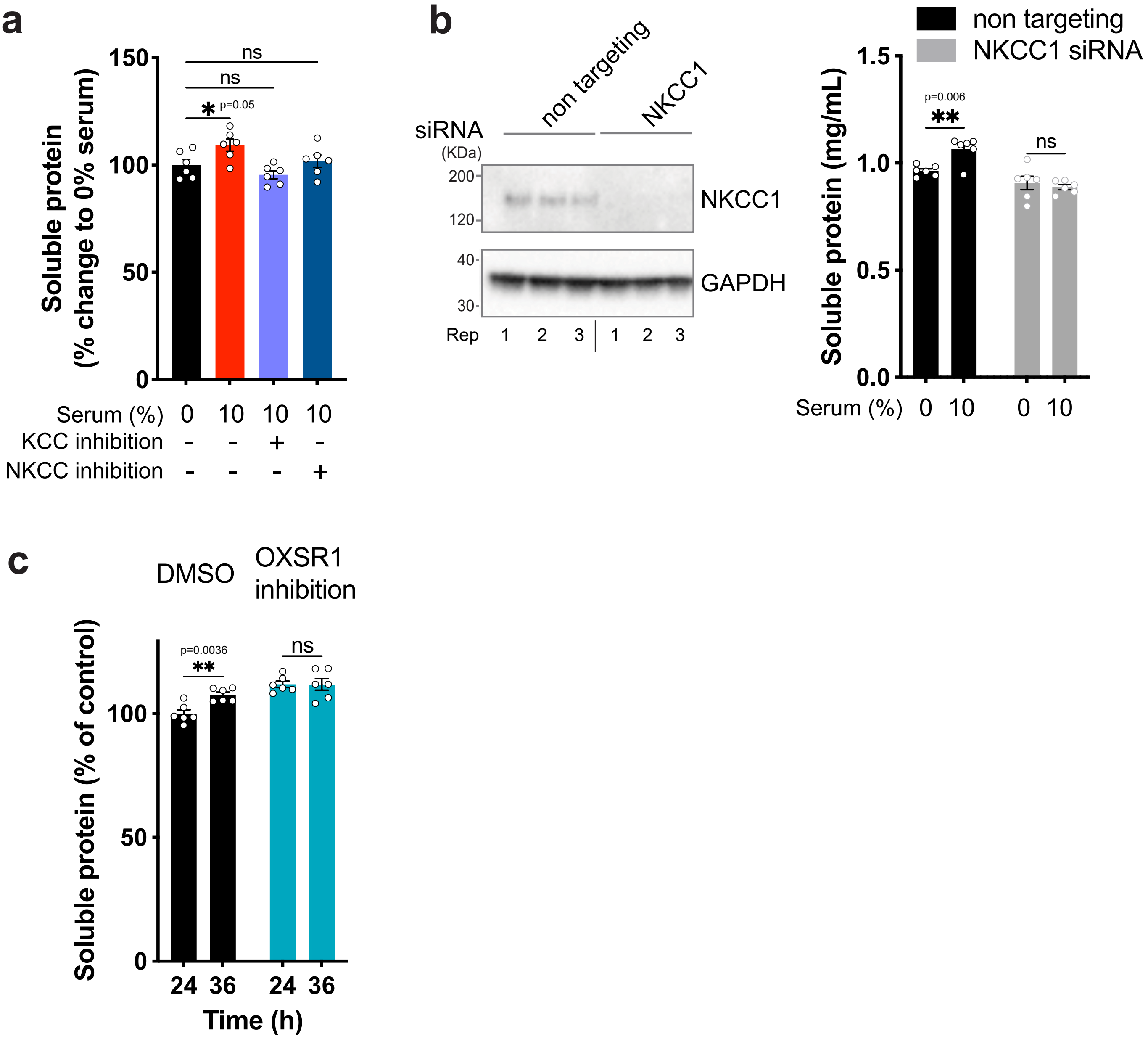

Supplementary Fig. 7. **Effect of OXSR1-SLC12A inhibition on soluble protein.**

a % change in soluble protein abundance upon treatment with the indicated stimuli for 4 h (n=6). Statistical test used was 2-way ANOVA and Dunnet MCT. **b** Left: immunoblot showing knock down efficiency of siRNA against NKCC1 (n=3). GAPDH was used as loading control. Right: soluble protein abundance upon 10% serum stimulation for 4 h in serum-starved fibroblasts transfected with non-targeting siRNA pool or siRNA against NKCC1. Statistical test used was two-way ANOVA with Sidak's MCT. **c** Soluble protein abundance at peak and trough of protein rhythms in fibroblasts ± 30 μM of OXSR1 inhibitor closantel (n=6). Closantel was added at time 0h. Data were normalised to control at 24 h. Statistical test used was two-way ANOVA with Sidak's MCT.

Data are presented as mean ± SEM. Cell type used was lung fibroblasts in (**a-b**) and cardiac fibroblasts in (**c**).

Supplementary Fig. 8

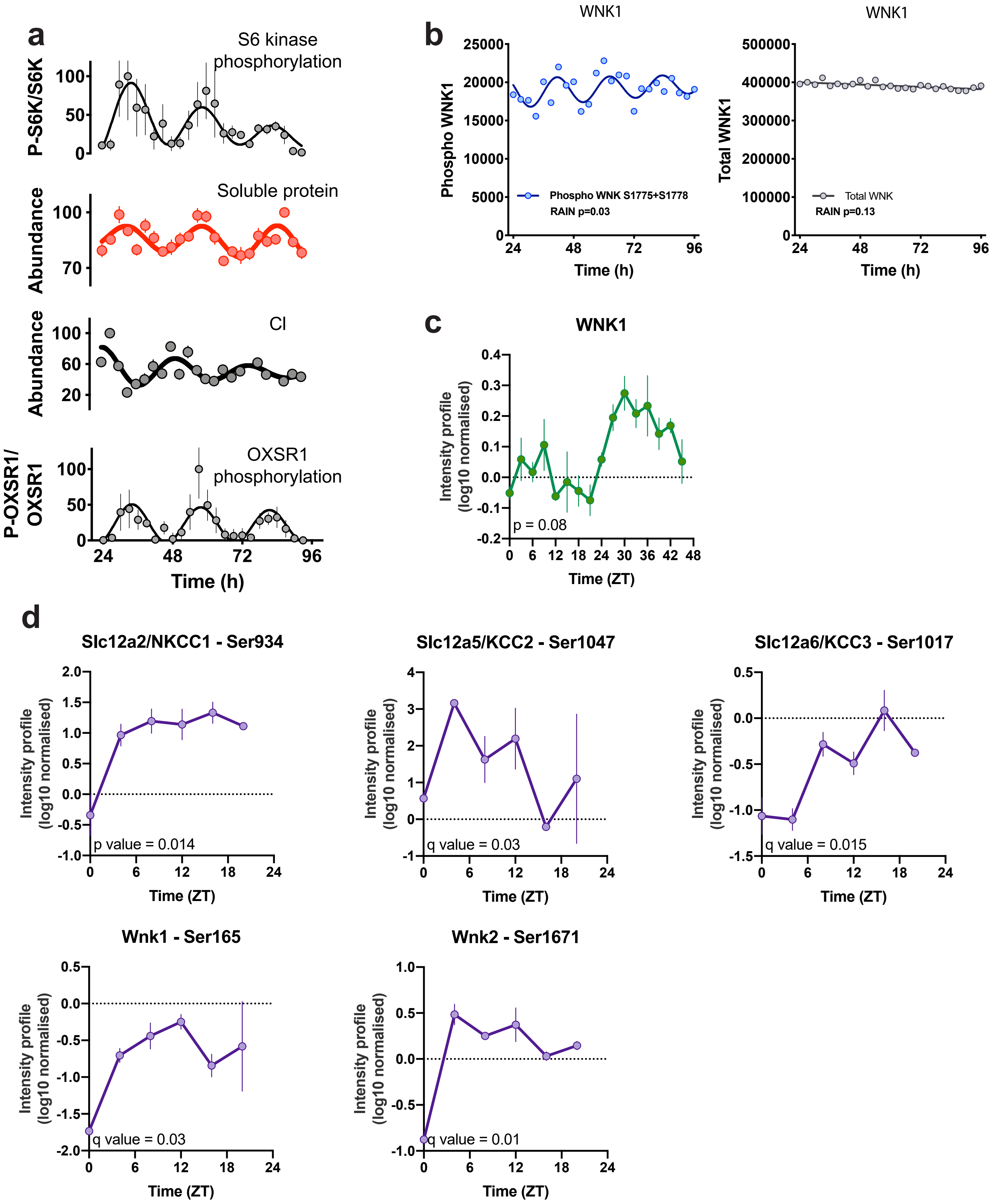

Supplementary Fig. 8. **Rhythms in the WNK-OXSR1 pathway.**

a Composite figure summarizing S6 Kinase, soluble protein, Cl, and OXSR1 rhythms, and phase relationship (n=3). Intensity profiles (phosphorylation) of indicated proteins taken from Wong *et al.*[57] (**b**) (n=3), Robles *et al.*, 2017[12] (**c**) (n=3), and Brüning *et al.*, 2019[30] (**d**) (n=3). Data are presented as mean ± SEM, p and q values are reported from the original papers.

Supplementary Fig. 9

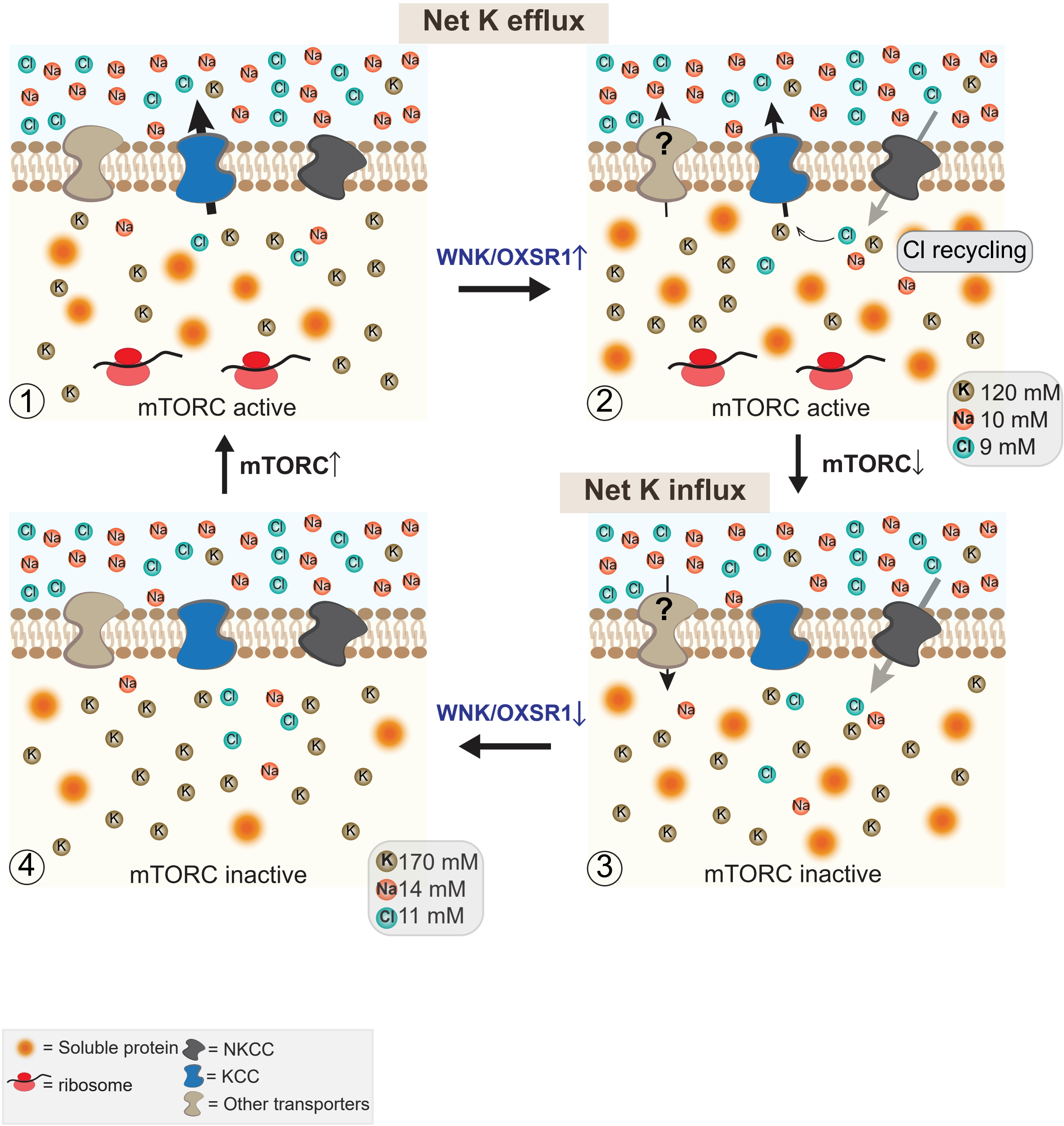

Supplementary Fig. 9. **Working model.**

Schematic of working model, from top left: (1) mTORC activation facilitates increased macromolecular crowding in the cytosol, whose osmotic potential is buffered by electroneutral KCl export; (2) increased crowding and reduced intracellular Cl^- concentration stimulates increased NKCC activity *via* WNK/OSXR1 allowing Cl^- recycling to sustain KCC-mediated K^+ efflux; (3) mTORC inactivation elicits a fall in cytosolic protein which is buffered by net K^+ influx *via* NKCC and other transporters; until (4) a new steady state is achieved without any net K^+ transport. Whilst SLC12A family members clearly contribute to this osmotic buffering system, other transporters are likely involved.

Supplementary Fig. 10

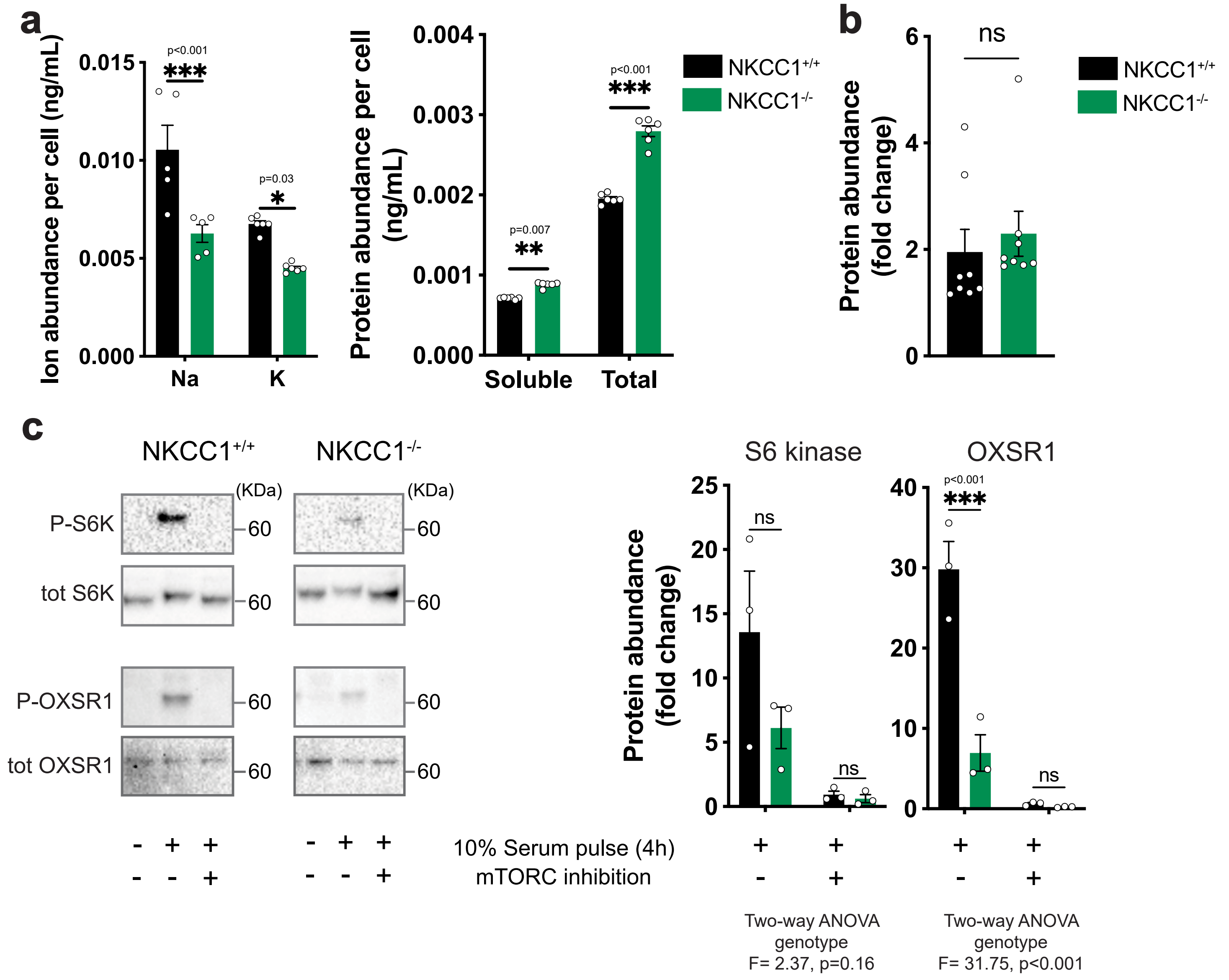

Supplementary Fig. 10. **Genetic ablation of NKCC1 affects ion/protein ratio in fibroblasts.**
a Ion and protein abundance per cell in $NKCC1^{+/+}$ and $NKCC1^{-/-}$ fibroblasts (n=6). **b** Change in soluble protein abundance upon 10% serum stimulation for 4 h (n=6). **c** Representative immunoblots and quantification of S6 Kinase and OXSR1 phosphorylation upon serum stimulation (4 h) in $NKCC1^{+/+}$ and $NKCC1^{-/-}$ fibroblasts (n=3).
Mean ± SEM shown throughout. Statistical tests used were two-way ANOVA and Sidak MCT (**a, c**) and two-tailed Student t-test (**b**). Cell type used was lung fibroblasts.

Supplementary Fig. 11

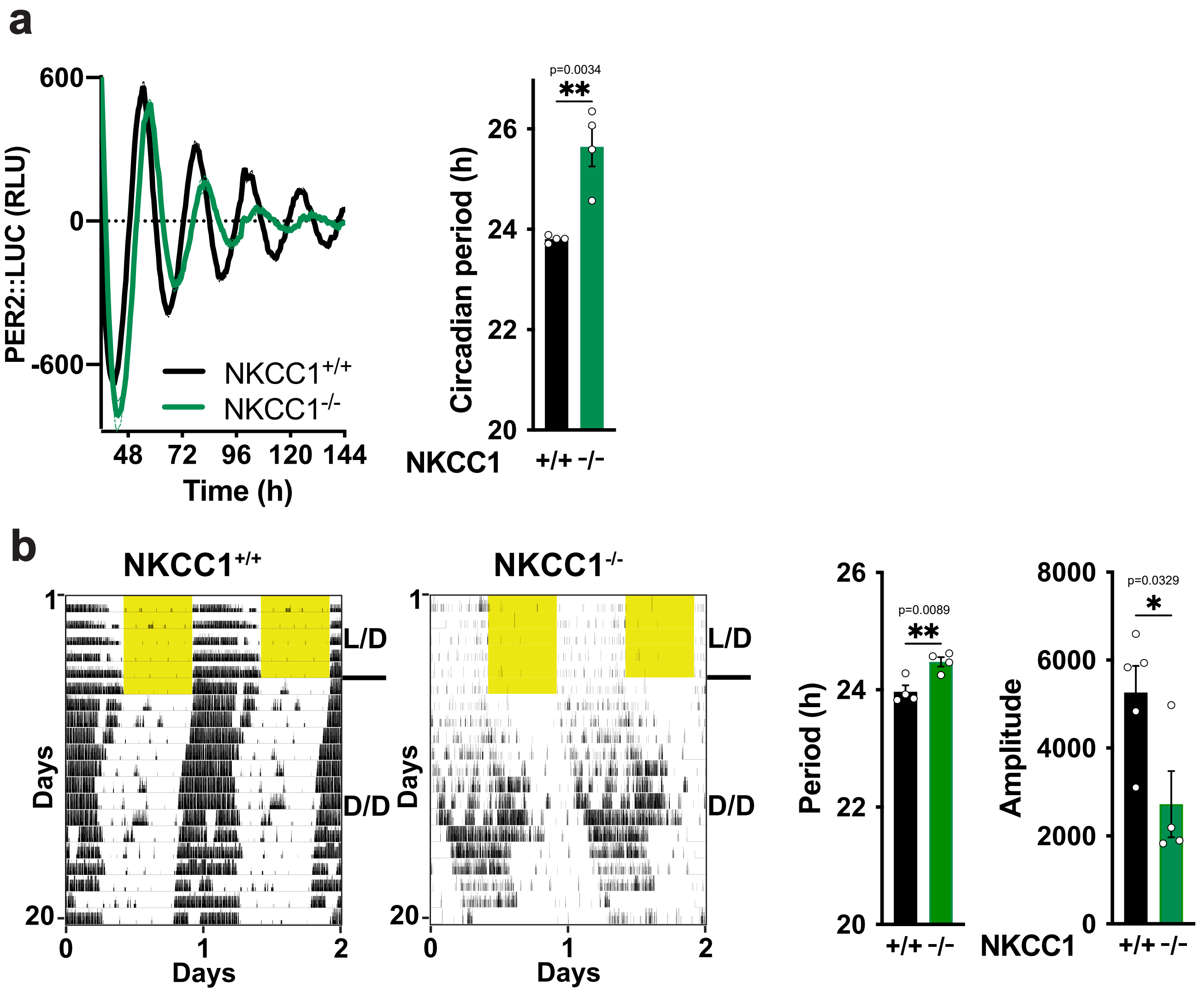

Supplementary Fig. 11. **Genetic ablation of NKCC1 affects cellular and organismal circadian rhythms.**

a Detrended PER2::LUC traces and circadian period quantification upon genetic ablation of NKCC1 (n=4) in cardiac fibroblasts. **b** Representative double-plotted actograms of NKCC1$^{+/+}$ and NKCC1$^{-/-}$ mice under light/dark cycles (L/D) and constant darkness (D/D), with circadian period and amplitude of wheel running activity under D/D (n=4 males per genotype). Yellow shading indicates lights ON.

Mean ± SEM shown throughout. Statistical tests used were two-tailed Student t-test.

Supplementary Fig. 12

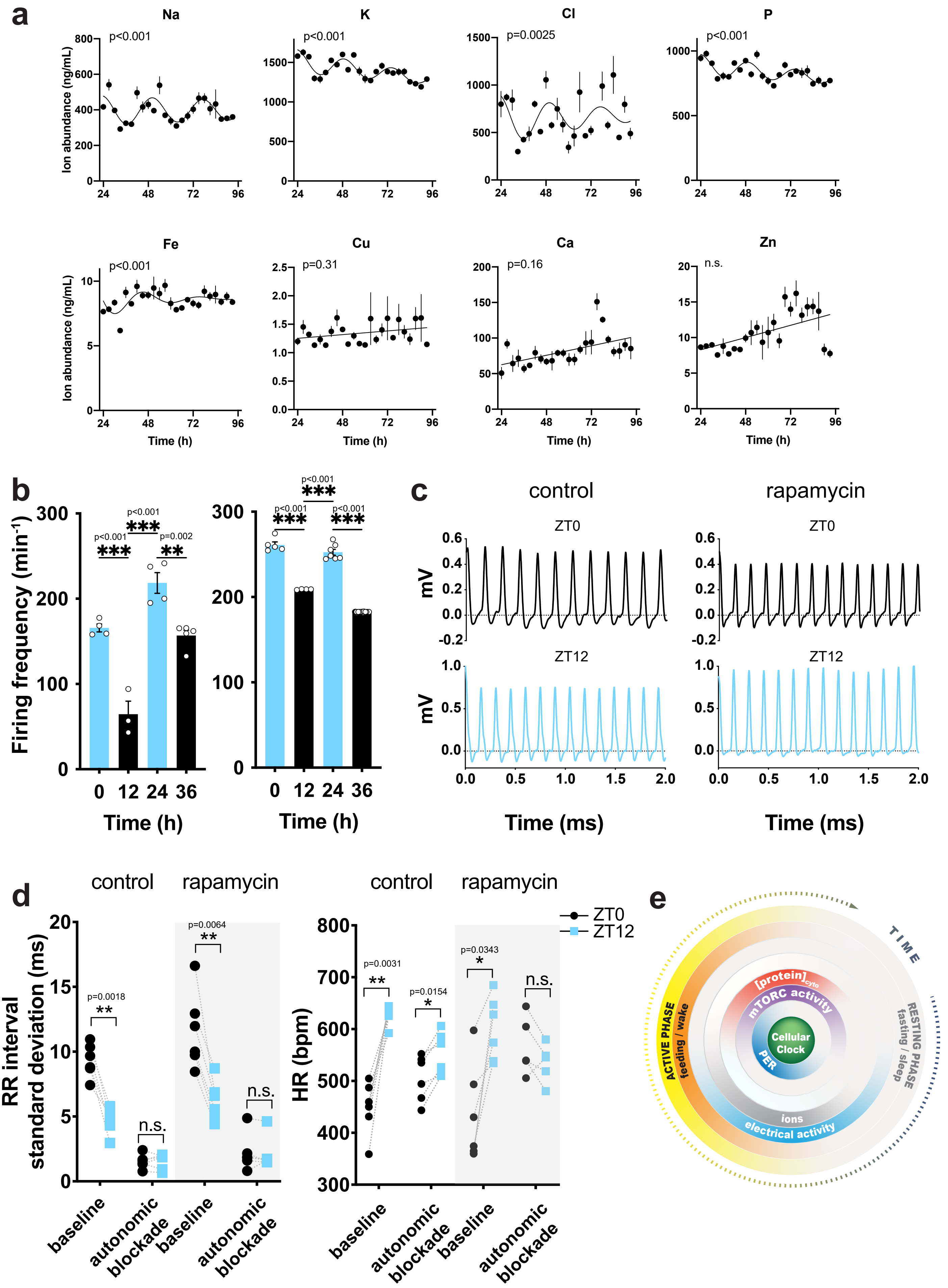

Supplementary Fig. 12. **Ion rhythms in cardiomyocytes and firing frequency.**

a ICP-MS quantification of biologically-relevant ions in primary cardiomyocytes (n=4). p-values indicate comparison between a damped cosine wave fit compared with a straight-line (null hypothesis = no rhythm). **b** Firing frequency of primary cardiomyocytes at the peak and trough of ion rhythms in two more independent biological replicates (mean values from active electrodes are presented). Statistical significance was calculated using one-way ANOVA with Tukey's test.

c Representative traces of *ex vivo* Langendorff recordings (monophasic action potentials) from control or rapamycin-treated mice at indicated times (n=6 for all but rapamycin autonomic blockade for which n=5). **d** RR interval standard deviation and Heart rate (HR) before and after autonomic blockade in control and rapamycin-treated mice (n=6). Reduction in RR interval standard deviation confirms the effect of autonomic blockade. Note that this does not affect the time-of-day variation in HR in control mice but only in the rapamycin-treated group. **e** Summary cartoon of the temporal relationships for *ex vivo* measurements with respect to gene expression and behaviour *in vivo*.

Data are presented as mean ± SEM. Statistical tests used were two-way ANOVA and Sidak MCT (**d**, RR) and mixed effect analysis and Sidak MCT (**d**, HR).

Ion	Extracellular concentration	Intracellular concentration (IC)	%change (IC)	Peak (IC)	Trough (IC)
**K**	4.5 mM	145 mM	~30%	~170 mM	~120 mM
**Na**	145 mM	12 mM	~40%	~14 mM	~10 mM
**Cl**	115 mM	10 mM	~20%	~11 mM	~9 mM

Supplementary Table 1. **Intracellular and extracellular ion concentrations in mammalian cells.**

Intracellular and extracellular ion concentrations in mammalian cells from[16]. Calculation of predicted intracellular concentration at peak and trough of ion rhythms (% change calculated from data in Fig. 2B).

Ion	Concentration (mM)	
	**Cardiac fibroblasts**	**Cardiac myocytes**
**Na**	94.07	88.51
**P**	88.99	137.89
**Cl**	113.39	114.83
**K**	114.55	183.45
**Ca43**	13.27	10.37
**Ca44**	10.99	11.27
**Cr**	0.06	0.11
**Mn**	0.02	0.02
**Fe**	0.53	0.78
**Co**	0.00	0.00
**Ni**	0.03	0.03
**Cu63**	0.10	0.14
**Cu65**	0.10	0.14
**Zn**	0.43	0.84
**Pb**	0.00	0.00

Supplementary Table 2. **Imputed values for ion concentrations in cardiac fibroblasts and cardiac myocytes.** Values were calculated from ICP-MS data assuming an average cell volume of 3.5 pL (80.000 cells lysed in 110 mM of 5% HNO_3). Data are from whole cell lysates and include ions stored into organelles.

References

102. Seliger, H. H. & McElroy, W. D. THE COLORS OF FIREFLY BIOLUMINESCENCE: ENZYME CONFIGURATION AND SPECIES SPECIFICITY. *Proc. Natl. Acad. Sci. U. S. A.* **52**, 75–81 (1964).

103. Adair, G. S. A Theory of Partial Osmotic Pressures and Membrane Equilibria, with Special Reference to the Application of Dalton's Law to Haemoglobin Solutions in the Presence of Salts. *Proc. R. Soc. A Math. Phys. Eng. Sci.* **120**, 573–603 (1928).

104. Halle, B. Protein hydration dynamics in solution: a critical survey. *Philos. Trans. R. Soc. London. Ser. B Biol. Sci.* **359**, 1207–1224 (2004).

105. Laage, D., Elsaesser, T. & Hynes, J. T. Water Dynamics in the Hydration Shells of Biomolecules. *Chem. Rev.* **117**, 10694–10725 (2017).

106. Marcus, Y. Effect of ions on the structure of water. *Pure Appl. Chem.* **82**, 1889–1899 (2010).

107. Ross, P. D. & Minton, A. P. Analysis of non-ideal behavior in concentrated hemoglobin solutions. *J. Mol. Biol.* **112**, 437–452 (1977).
