# Supplementary material for: Compensatory ion transport buffers daily protein rhythms to regulate osmotic balance and cellular physiology
Source: Nat Commun. 2021 Oct 15;12:6035. doi: 10.1038/s41467-021-25942-4 (PMC8520019; doi:10.1038/s41467-021-25942-4)
Supplement: Supplementary file 5 — Description of additional supplementary files [file 41467_2021_25942_MOESM5_ESM.docx]

Description of additional supplementary information

Title: Supplementary video 1.

Description: Tracking and effective diffusion of quantum dots at peak and trough of protein rhythms.

Title: Supplementary video 2.

Description: Tracking and effective diffusion of quantum dots upon challenge with media at different osmolality.
