# Supplementary material for: Compensatory ion transport buffers daily protein rhythms to regulate osmotic balance and cellular physiology
Source: Nat Commun. 2021 Oct 15;12:6035. doi: 10.1038/s41467-021-25942-4 (PMC8520019; doi:10.1038/s41467-021-25942-4)
Supplement: Supplementary file 6 — Reporting Summary [file 41467_2021_25942_MOESM6_ESM.pdf]

nature research

Corresponding author(s): John O'Neill

Last updated by author(s): Jun 30, 2021

Reporting Summary

Nature Research wishes to improve the reproducibility of the work that we publish. This form provides structure for consistency and transparency in reporting. For further information on Nature Research policies, see our Editorial Policies and the Editorial Policy Checklist.

Statistics

For all statistical analyses, confirm that the following items are present in the figure legend, table legend, main text, or Methods section.

n/a	Confirmed	
☐	☒	The exact sample size (*n*) for each experimental group/condition, given as a discrete number and unit of measurement
☐	☒	A statement on whether measurements were taken from distinct samples or whether the same sample was measured repeatedly
☐	☒	The statistical test(s) used AND whether they are one- or two-sided<br>*Only common tests should be described solely by name; describe more complex techniques in the Methods section.*
☒	☐	A description of all covariates tested
☐	☒	A description of any assumptions or corrections, such as tests of normality and adjustment for multiple comparisons
☐	☒	A full description of the statistical parameters including central tendency (e.g. means) or other basic estimates (e.g. regression coefficient) AND variation (e.g. standard deviation) or associated estimates of uncertainty (e.g. confidence intervals)
☐	☒	For null hypothesis testing, the test statistic (e.g. *F*, *t*, *r*) with confidence intervals, effect sizes, degrees of freedom and *P* value noted<br>*Give P values as exact values whenever suitable.*
☒	☐	For Bayesian analysis, information on the choice of priors and Markov chain Monte Carlo settings
☒	☐	For hierarchical and complex designs, identification of the appropriate level for tests and full reporting of outcomes
☒	☐	Estimates of effect sizes (e.g. Cohen's *d*, Pearson's *r*), indicating how they were calculated

Our web collection on statistics for biologists contains articles on many of the points above.

Software and code

Policy information about availability of computer code

Data collection	Bioluminescence data were collected using Optomorph (7.7.110) or AndorSolis (32-bit 4.32.30000.0). Confocal images were acquired using LeicaSP8 microscope and LASX software and cell volume analysis was performed with Imaris 8.2 (Oxford Instruments). ICP-MS data were collected and analyzed using Syngistix version 1.1. Estimation of the effective diffusion coefficient of QDs was performed using commercially available software (Fiji (with ImageJ 1.52t and Thunderstorm plugin), MATLAB R2020a (with msdanalyzer library)), and using custom codes (deposited in https://github.com/deriverylab/Stangherlin2021). Proteomics data were analysed with MaxQuant (Cox and Mann) with the integrated Andromeda search engine (v.1.6.6.0). Gene enrichment analysis was performed with PANTHER version 15.0 Released 2020-02-14. Wheel running experiments were performed using ClockLab3 and analysed with ClockLab Analysis software (6.1.01) both from Actimetrics.
Data analysis	Graphpad Prism 9 was used for all statistical analyses.

For manuscripts utilizing custom algorithms or software that are central to the research but not yet described in published literature, software must be made available to editors and reviewers. We strongly encourage code deposition in a community repository (e.g. GitHub). See the Nature Research guidelines for submitting code & software for further information.

Data

Policy information about availability of data

All manuscripts must include a data availability statement. This statement should provide the following information, where applicable:

- Accession codes, unique identifiers, or web links for publicly available datasets
- A list of figures that have associated raw data
- A description of any restrictions on data availability

The data supporting the findings of this study are available in the main article and supplementary files or from the corresponding author upon reasonable request. Source data are provided with this paper as Source Data File. The original mass spectra and search engine files used in this study have been deposited in the public

proteomics repository MassIVE (https://massive.eusd.edu) and are accessible at ftp://massive.eusd.edu/MSV000087673.
The codes used for detection, tracking, and analysis of quantum dots are available for download through GitHub (https://github.com/deriverylab/Stangherlin2021).

Field-specific reporting

Please select the one below that is the best fit for your research. If you are not sure, read the appropriate sections before making your selection.

☒ Life sciences ☐ Behavioural & social sciences ☐ Ecological, evolutionary & environmental sciences

For a reference copy of the document with all sections, see nature.com/documents/nr-reporting-summary-flat.pdf

Life sciences study design

All studies must disclose on these points even when the disclosure is negative.

Sample size	Numbers of biological replicates were chosen based on preliminary experiments, so that an effect size of at least 10% could be detected between experimental groups for α=0.05 and β=0.9.
Data exclusions	Some values from the ICP-MS analyses were excluded using the ROUT method in GraphPad (Q=0.1%, highest stringency).
Replication	For cell-based experiments, replication was performed by using at least three biological replicates (two for MEA experiments in Fig.4d) and the experiments confirmed at least twice. For osmometry, and measurement of QDs in solution, experiments performed on one occasion with multiple technical replicates. For experiments involving mice, careful consideration was given to experimental design to optimization of animal use. Therefore the experiments were performed on one occasion with at least 3 mice per experimental group. The exact n is reported in the figure legends.
Randomization	For cell experiments, samples were randomly allocated to each group, and subsequently handled and processed identically at each time point. No potential covariates could be identified that required any additional controls.
Blinding	Investigators were not blinded during data collection as all the replicates (control and treatments) were collected/extracted and/or analyzed using the same procedure within each experiment (objective measurements). Where practical, investigators were ignorant to the hypothesis, sampling methodology and identity of biological samples, e.g., ICP-MS measurements, protein mass spectrometry.

Reporting for specific materials, systems and methods

We require information from authors about some types of materials, experimental systems and methods used in many studies. Here, indicate whether each material, system or method listed is relevant to your study. If you are not sure if a list item applies to your research, read the appropriate section before selecting a response.

Materials & experimental systems

n/a	Involved in the study	
☐	☒	Antibodies
☐	☒	Eukaryotic cell lines
☒	☐	Palaeontology and archaeology
☐	☒	Animals and other organisms
☒	☐	Human research participants
☒	☐	Clinical data
☒	☐	Dual use research of concern

Methods

n/a	Involved in the study	
☒	☐	ChIP-seq
☒	☐	Flow cytometry
☒	☐	MRI-based neuroimaging

Antibodies

Antibodies used	Primary antibodies used were: Anti-OXSR1 (Abcam, ab224248), 1/1000; anti- Phospho-OXSR1 (Abcam, ab138655), 1/1000; anti S6K (Cell signalling, 2708), 1/2000; anti P-S6K (Cell signalling, 9205); anti-NKCC1 (Cell Signalling, 14581), 1/500; anti-GAPDH (Sigma, G9545), 1/2000; HRP-conjugated secondary antibody was anti-rabbit (Sigma-Aldrich, A6154).
Validation	All the antibodies are commercially available; representative blots are shown in their data-sheet as well as background references.<br>Anti-OXSR1 Abcam, ab22424: https://www.abcam.com/oxsr1-antibody-ab224248.html<br>Anti- Phospho-OXSR1, Abcam, ab138655: https://www.abcam.com/oxsr1-phospho-t185-antibody-ab138655.html<br>Anti S6K ,Cell signalling, 2708: https://www.cellsignal.co.uk/products/primary-antibodies/p70-s6-kinase-49d7-rabbit-mab/2708<br>Anti P-S6K ,Cell signalling, 9205: https://www.cellsignal.co.uk/products/primary-antibodies/phospho-p70-s6-kinase-thr389-Antibody/9205?_=1624775780747&Ntt=9205&tahead=true<br>Anti-NKCC1, Cell Signalling, 14581: https://www.cellsignal.co.uk/products/primary-antibodies/nkcc1-antibody-rodent-specific/14581<br>Anti-GAPDH, Sigma, G9545: https://www.sigmaaldrich.com/GB/en/product/sigma/g9545?gclid=CjwKCAjwoNuGBhA8EiwAFxomA5-faf8UoYnUyDWJ5VqNHZhsulTu6eZ-AUasvHI0-VHCbcBQvA3FchoCxEYQAvD_BwE

In addition, for anti-total OXSR1 the correct band was identified by western blot of mouse fibroblasts lysates where OXSR1 expression was abolished by siRNA (no band was present at the expected molecular weight). For anti-phospho OXSR1 the correct band was identified by treating the sample with a phosphatase. For Specificity of the anti-NKCC1 antibody was tested by western blot in NKCC1-/- mouse fibroblast cell lines, no band was present at the expected molecular weight.

Eukaryotic cell lines

Policy information about cell lines

Cell line source(s)	The cell lines used in this study were isolated in-house from tissues taken from C57BL/6J mice, that were homozygous for the PER2::LUC transgene (Yoo et al., PNAS, 2004) and otherwise wild type; or else also homozygous for the tau mutation (CK1εtau/tau, Meng et al., Neuron, 2008) or homozygous null for NKCC1 (Kim et al., Am J Renal Physiol., 2008). Genotype was confirmed by PCR.
Authentication	N/A - Cell lines were derived in-house for this study.
Mycoplasma contamination	Primary cell lines were derived in-house from mice bred in a specified pathogen free barrier facility. Lung and cardiac fibroblasts were tested for mycoplasma via a PCR-based method, several passages after isolation. All cell lines tested negative.
Commonly misidentified lines (See ICLAC register)	No commonly misidentified cell lines were used in this study

Animals and other organisms

Policy information about studies involving animals; ARRIVE guidelines recommended for reporting animal research

Laboratory animals	Male, C57BL/6J mice aged 10-13 weeks at the start of study, purchased from Charles River (UK), were used for ECG and Langendorff studies. Housing conditions: temperature 22C+/-2C; humidity 52%+/-7%; food and water provided ad libitum; ambient datk:light cycles is defined by experiment in the methods section.<br><br>8-12 week old male mice were used for the wheel running activity experiment. NKCC1+/+ and NKCC1-/- mice were back-crossed into a transgenic mouse line expressing the circadian clock reporter PER2::LUC (Yoo et al. PNAS, 2004).<br>Mice were housed at 19-23 degrees celsius and 45-65% humidity; food and water were provided ad libitum; ambient light/dark cycles for behavioral experiments are defined in the methods sections and in the figure legends.<br><br>Primary lung fibroblasts were obtained from 6-8 week old male WT PER2::LUC and TAU PER2::LUC mice housed at 19-23 degrees Celsius and 45-65% humidity; food and water were provided ad libitum; ambient light/dark cycles 12h/12h. Primary cardiac fibroblasts were obtained from mixed sex P2-P3 neonatal mice.
Wild animals	The study did not involve wild animals.
Field-collected samples	The study did not involve samples collected from the field.
Ethics oversight	All animal experiments were licensed under 1986 Home Office Animal Procedures Act (UK) and carried out in accordance with local animal welfare committee guidelines; either the LMB Animal Welfare and Ethical Review Body or, for Langendorff experiments and in vivo HR measurements, the University of Manchester Animal Welfare and Ethical Review Body.

Note that full information on the approval of the study protocol must also be provided in the manuscript.
