# Supplementary material for: Compensatory ion transport buffers daily protein rhythms to regulate osmotic balance and cellular physiology
Source: Nat Commun. 2021 Oct 15;12:6035. doi: 10.1038/s41467-021-25942-4 (PMC8520019; doi:10.1038/s41467-021-25942-4)
Supplement: Supplementary file 7 — Source data [file 41467_2021_25942_MOESM7_ESM.zip › Uncropped blots.pdf]

Uncropped blots - 1

Original blots for Fig. 1a

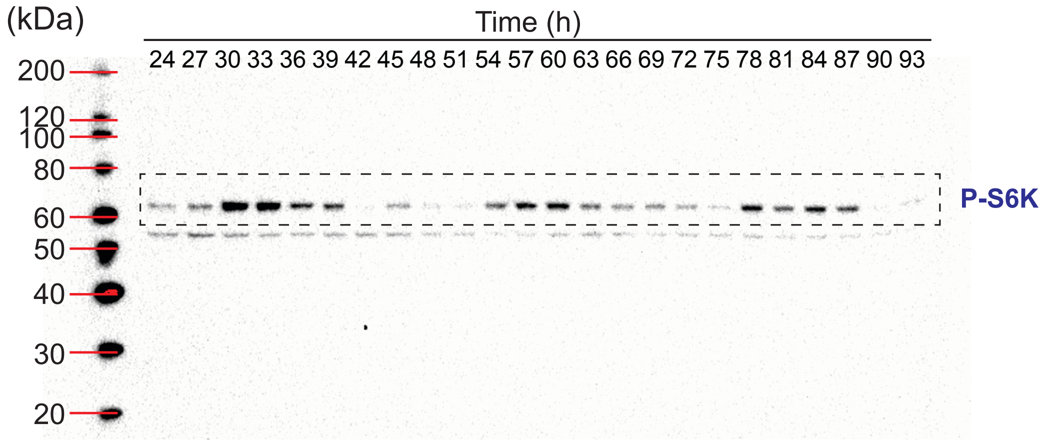

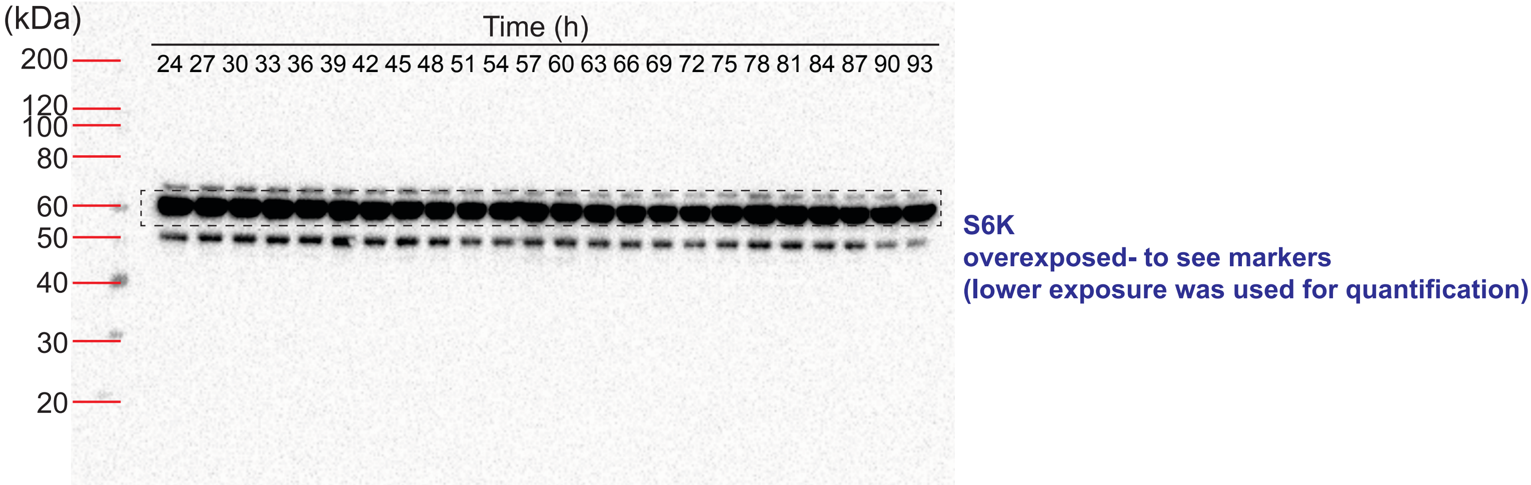

anti S6K antibody used was from Cell Signalling (2708)
anti P-S6K antibody used was from Cell Signalling (9205)

Uncropped blots - 2

Original blots for Fig. 3b

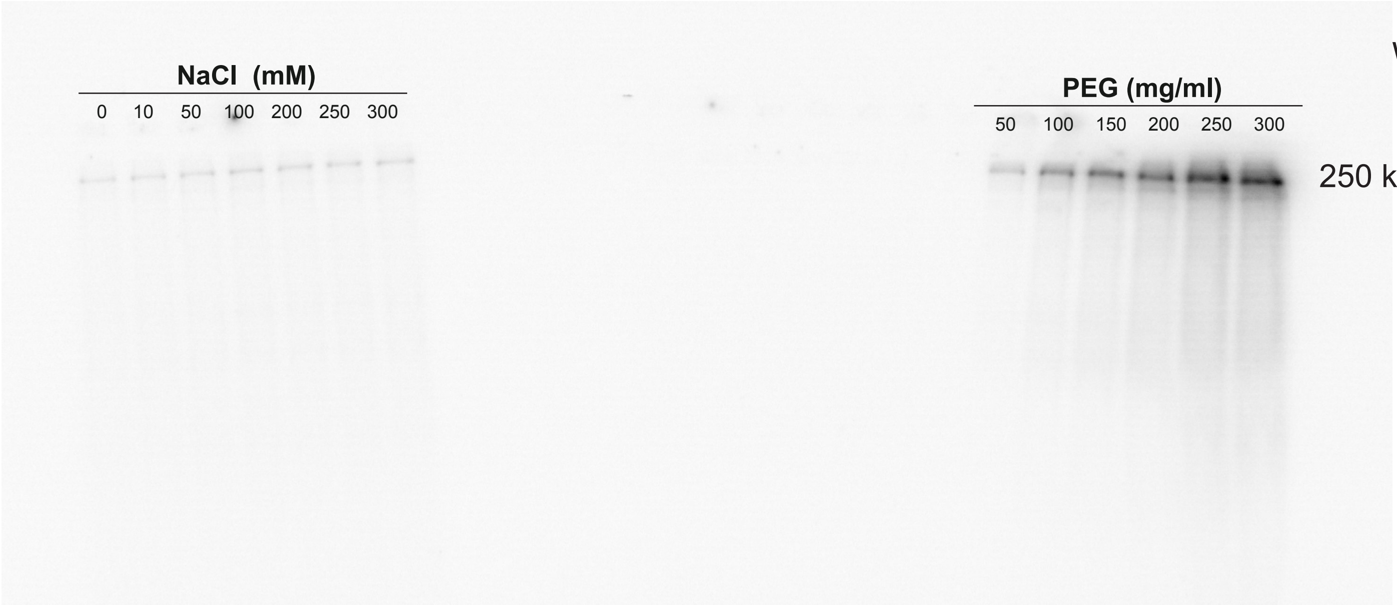

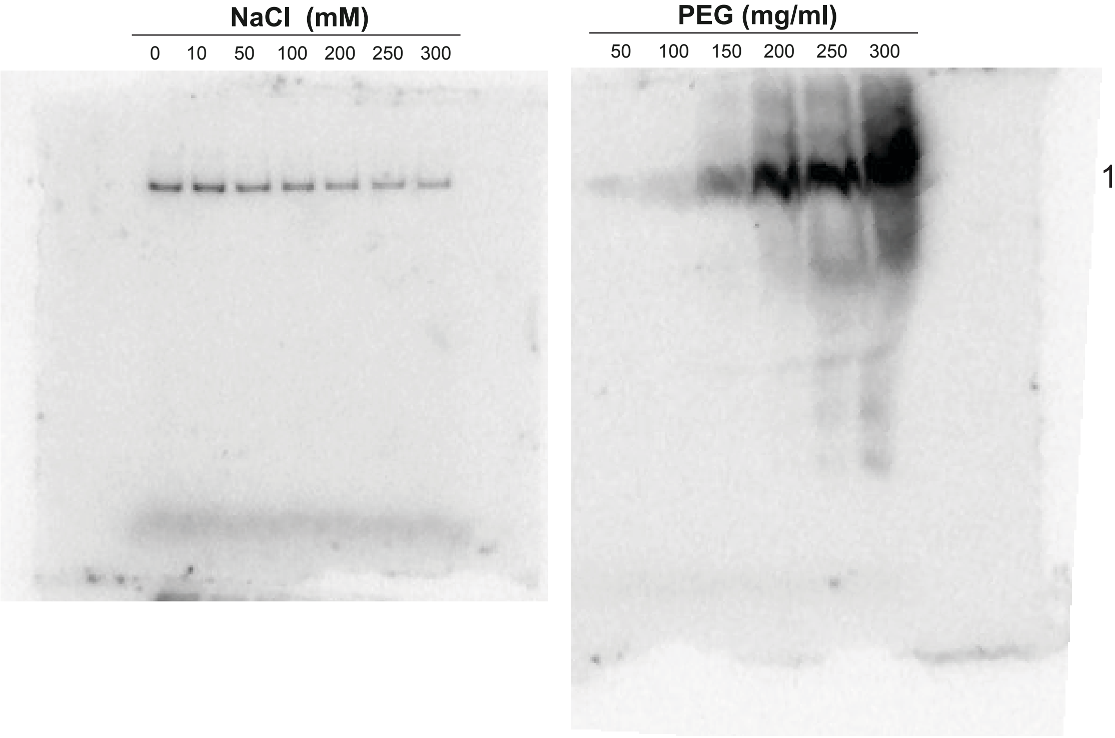

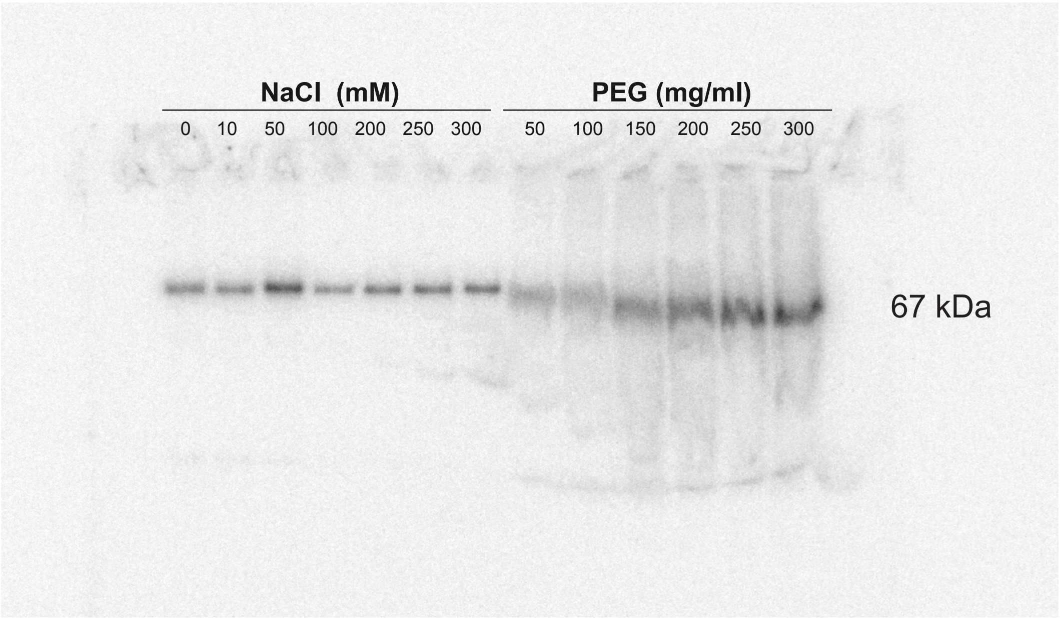

Uncropped blots - 3

(a) Original blots for Fig. 3c

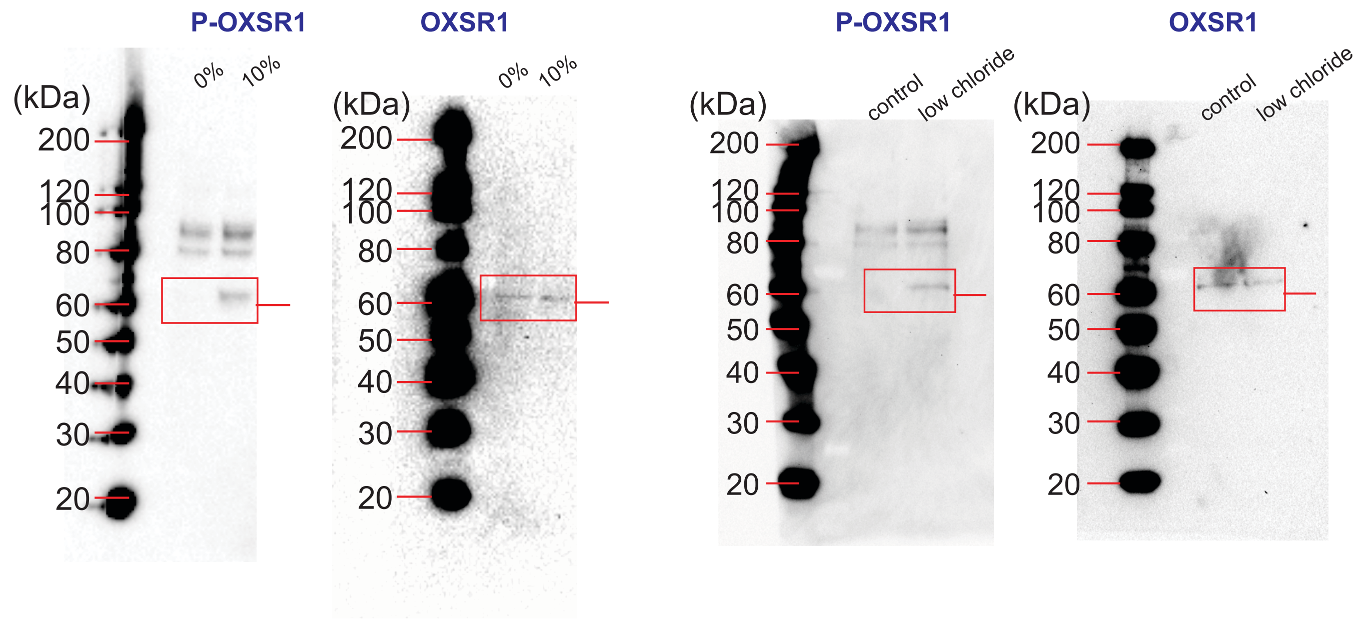

(b) Original blots for Fig. 3d

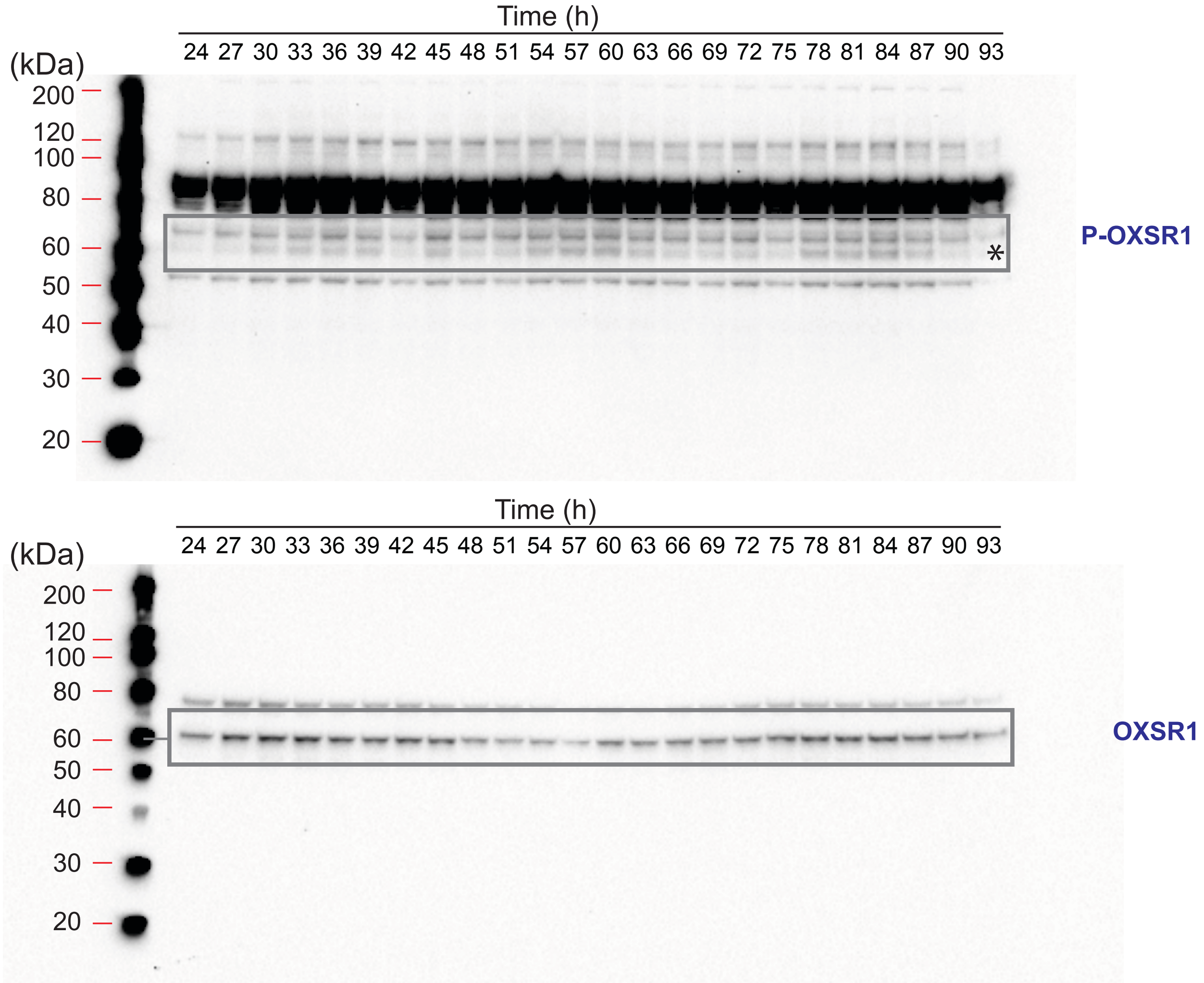

Anti-OXSR1 was from Abcam (ab224248),
anti- Phospho-OXSR1 was from Abcam (ab138655)

Uncropped blots - 4

Original blots for Suppl Fig. 7b

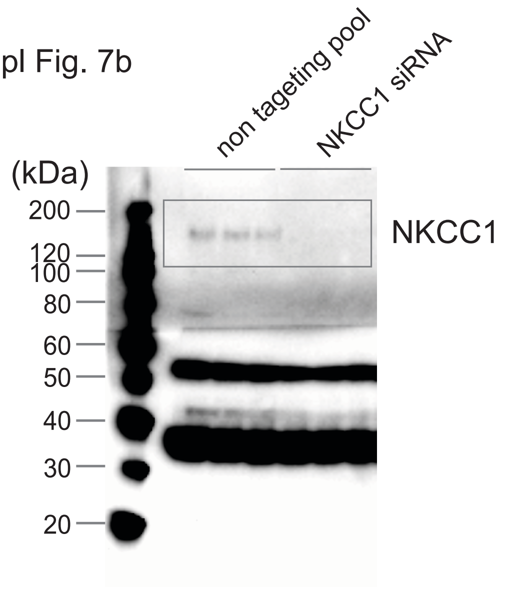

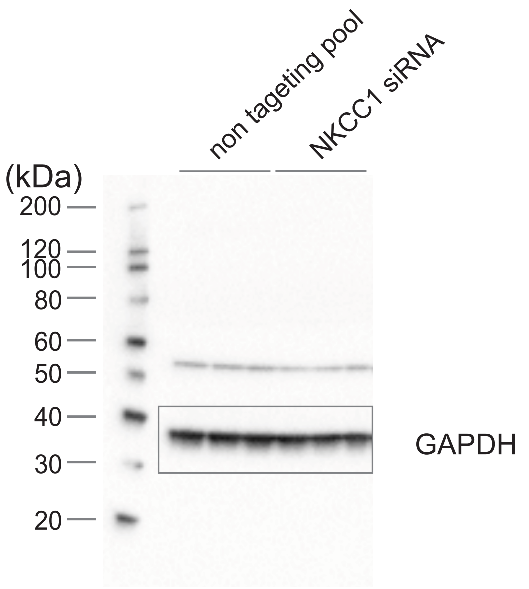

Original blots for Suppl Fig. 10c

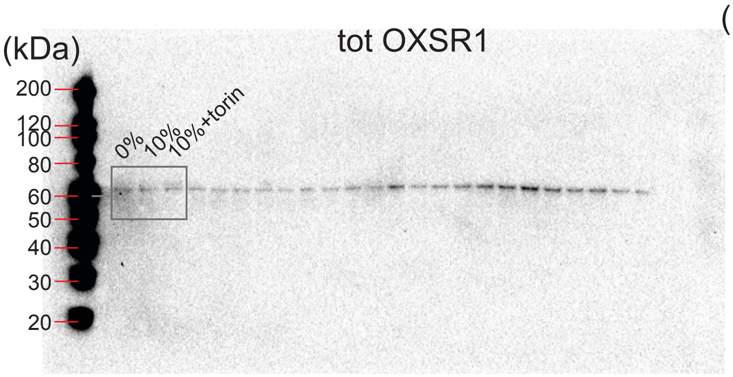

Anti-OXSR1 was from Abcam (ab224248)

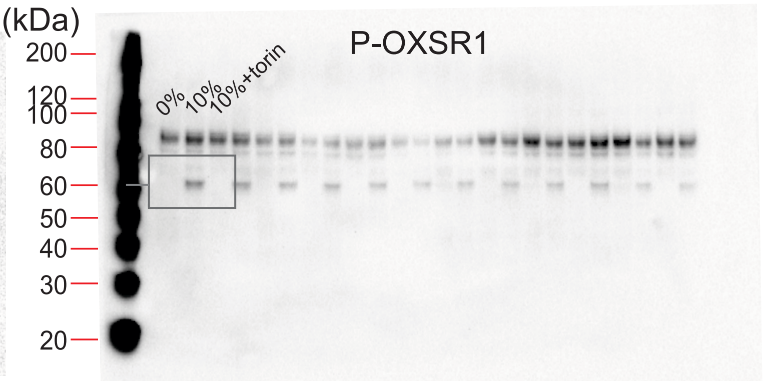

anti- Phospho-OXSR1 was from Abcam (ab138655)

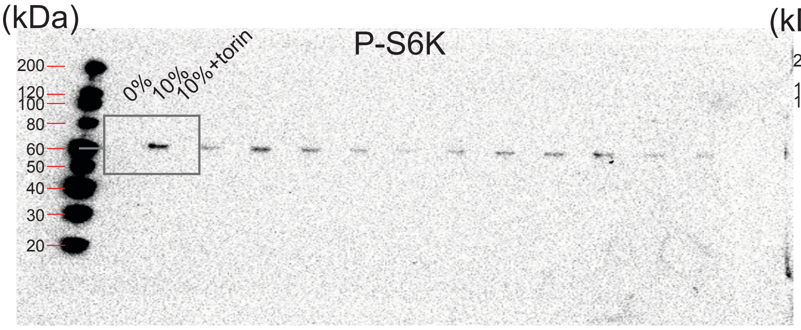

anti P-S6K antibody used was from Cell Signalling (9205)

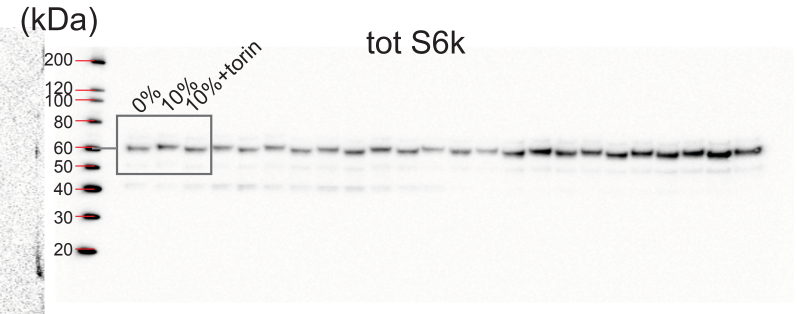

anti S6K antibody used was from Cell Signalling (2708)
